# Mistimed sleep and waking activity in humans disrupts glucocorticoid signalling transcripts and SP1, but not plasma cortisol rhythms

**DOI:** 10.3389/fphys.2022.946444

**Published:** 2022-08-17

**Authors:** Simon N. Archer, Carla S. Möller-Levet, Emma E. Laing, Derk-Jan Dijk

**Affiliations:** ^1^ Surrey Sleep Research Centre, Faculty of Health and Medical Sciences, University of Surrey, Guildford, United Kingdom; ^2^ Bioinformatics Core Facility, Faculty of Health and Medical Sciences, University of Surrey, Guildford, United Kingdom; ^3^ School of Biosciences and Medicine, Faculty of Health and Medical Sciences, University of Surrey, Guildford, United Kingdom; ^4^ UK Dementia Research Institute Care Research and Technology Centre, Imperial College London and the University of Surrey, Guildford, United Kingdom

**Keywords:** forced desynchrony, circadian, transcriptome, whole blood, glucocorticoid, cortisol, SP1

## Abstract

Cortisol is a robust circadian signal that synchronises peripheral circadian clocks with the central clock in the suprachiasmatic nucleus *via* glucocorticoid receptors that regulate peripheral gene expression. Misalignment of the cortisol rhythm with the sleep–wake cycle, as occurs in shift work, is associated with negative health outcomes, but underlying molecular mechanisms remain largely unknown. We experimentally induced misalignment between the sleep–wake cycle and melatonin and cortisol rhythms in humans and measured time series blood transcriptomics while participants slept in-phase and out-of-phase with the central clock. The cortisol rhythm remained unchanged, but many glucocorticoid signalling transcripts were disrupted by mistimed sleep. To investigate which factors drive this dissociation between cortisol and its signalling pathways, we conducted bioinformatic and temporal coherence analyses. We found that glucocorticoid signalling transcripts affected by mistimed sleep were enriched for binding sites for the transcription factor SP1. Furthermore, changes in the timing of the rhythms of *SP1* transcripts, a major regulator of transcription, and changes in the timing of rhythms in transcripts of the glucocorticoid signalling pathways were closely associated. Associations between the rhythmic changes in factors that affect SP1 expression and its activity, such as STAT3, EP300, HSP90AA1, and MAPK1, were also observed. We conclude that plasma cortisol rhythms incompletely reflect the impact of mistimed sleep on glucocorticoid signalling pathways and that sleep–wake driven changes in SP1 may mediate disruption of these pathways. These results aid understanding of mechanisms by which mistimed sleep affects health.

## 1 Introduction

Circadian rhythms are present in nearly every tissue and organ and are synchronised to the external world by the central circadian clock in the hypothalamic suprachiasmatic nuclei (SCN). Rhythmicity in tissues/organs in the body is synchronised to the SCN clock via a mixture of neural and humoral signals, although rhythms in some tissues/organs such as liver, stomach, muscle, adipose, and non-SCN brain areas are also influenced by the fasting–feeding cycle and sleep–wake cycle (Mistlberger, 2020; Pilorz et al., 2020; Jha et al., 2021). Cortisol is a glucocorticoid hormone whose synthesis by the adrenal glands is regulated by the hypothalamo–pituitary–adrenal axis. Daily rhythms in cortisol are driven by the SCN acting on the paraventricular nucleus via either inhibitory GABAergic neurons (in nocturnal mammals) or excitatory glutamatergic neurons (in diurnal mammals) in the sub-paraventricular nucleus and via the dorsomedial nucleus. This regulates the release of corticotropin-releasing hormone, which stimulates the release of adrenocorticotropic hormone from the anterior pituitary that then controls the release of corticosterone from the adrenal cortex. The SCN also regulates the sensitivity of melanocortin receptors to adrenocorticotropic hormone via preautonomic neurons from the parvocellular region of the paraventricular nucleus (Buijs et al., 2003; Kalsbeek and Buijs, 2021). Cortisol is a robust circadian signal that peaks upon awakening, persists in the absence of a sleep–wake cycle, and prepares the body biochemically and metabolically for daily activity (Koop and Oster, 2021).

Cortisol synchronises peripheral clocks in many tissues/organs, including several areas of the brain, and can directly entrain and activate molecular components of the circadian clock, such as PERIOD and BMAL1 (ARNTL) (Balsalobre et al., 2000; Reddy et al., 2012; Cuesta et al., 2015). However, in addition to regulating the circadian molecular clock in peripheral tissues/organs, glucocorticoid has also been shown to regulate the expression of thousands of genes via activation of glucocorticoid receptors (GR) which then either interact directly with glucocorticoid response elements (GRE) in promoter regions or regulate gene expression indirectly by interacting with other proteins and transcription factors (Reddy et al., 2009; Yu et al., 2010; Polman et al., 2012; Oakley and Cidlowski, 2013; Ratman et al., 2013; Wilmot Roussel et al., 2013). Cortisol signals via GR, which are activated upon chaperone dissociation, to regulate gene expression either directly by binding to GRE to regulate transcription by the RNA polymerase 2 complex or indirectly by binding GRE to regulate expression in conjunction with other adjacent transcription factors (TFs) in composite regulation, and indirectly by interacting with other TFs in tethering. Non-genomic glucocorticoid signalling also suppresses inflammatory cytokine production via inhibition of MAP kinases and regulates components of cell cycle control (Roth et al., 2004; Berg et al., 2005; Oakley and Cidlowski, 2013; Ratman et al., 2013). It is via these direct and indirect gene expression signalling pathways that glucocorticoid regulates a range of physiological processes including inflammation (Gibbs et al., 2014; Hand et al., 2016), immune function (Diaz-Jimenez et al., 2021), and glucose and insulin homeostasis (Geer et al., 2014; Kuo et al., 2015), in addition to its role in the stress response (Mourtzi et al., 2021).

The cortisol rhythm has been shown to be robust against acute changes in environment and behaviour. Experimental protocols in which rest/activity cycles are misaligned with the SCN phase, as indexed by melatonin, have shown that the cortisol rhythm remains phase-locked to the melatonin rhythm and that its amplitude does not change significantly (Scheer et al., 2009; McHill et al., 2018). However, in some studies, the rhythms of cortisol have been shown to be affected. For example, a study of diurnal rhythms in police officers after consecutive night shifts found that over the course of 7 days of shifts the phase of the cortisol rhythm shifted by 33 min per day but amplitude did not change (Jensen et al., 2016). Similarly, in another study, the sleep schedule was gradually phase-advanced by 10 h over 5 days, and participants were exposed to either moderate (90–150 lux) or bright (10,000 lux) light during the day. Under such conditions, the cortisol rhythm advanced by 10 h and remained aligned to the sleep schedule under the bright light condition, but only advanced by 2 h in the moderate light condition and reductions in amplitude were observed (Dijk et al., 2012). In a study where participants followed a 3-day rotating shift schedule for 29 days, cortisol amplitude was elevated in the initial days and rhythms became phase-delayed (Huang et al., 2021). In summary, most studies have shown modest reductions in mesor or amplitude of cortisol rhythms associated with shift work (see (Boivin et al., 2022) for review).

Circadian misalignment, i.e., abnormal timing of the rest–activity cycle relative to SCN rhythms, has been associated with negative health consequences that are associated with, for example, insulin resistance and inflammation (Leproult et al., 2014), insulin sensitivity (Eckel et al., 2015), and glucose tolerance (Morris et al., 2015), but underlying molecular mechanisms are not well characterised. Circadian misalignment most commonly occurs due to night shift schedules, which are associated with a range of adverse health effects that include sleep disturbance, impaired cognitive function, cardiometabolic disorders, hypertension, metabolic syndrome, impaired glucose tolerance, diabetes, cancer, and depression (see (Boivin et al., 2022) for review). Shift work can lead to circadian rhythms of cortisol that are misaligned with respect to the shifted rest–activity cycle, and this misalignment of cortisol signalling to the periphery may underlie some of the observed disruption to physiology and health. However, cortisol regulates peripheral circadian rhythms by signalling via a network of molecular pathways and it is not known how these signalling pathways are affected by circadian misalignment.

In this study, our aim was to investigate the effect of experimentally induced misalignment between the rest–activity cycle and SCN-driven rhythms such as cortisol and melatonin, on the temporal expression profiles of transcripts coding for molecular components of glucocorticoid signalling pathways.

## 2 Methods

**FIGURE 1:**
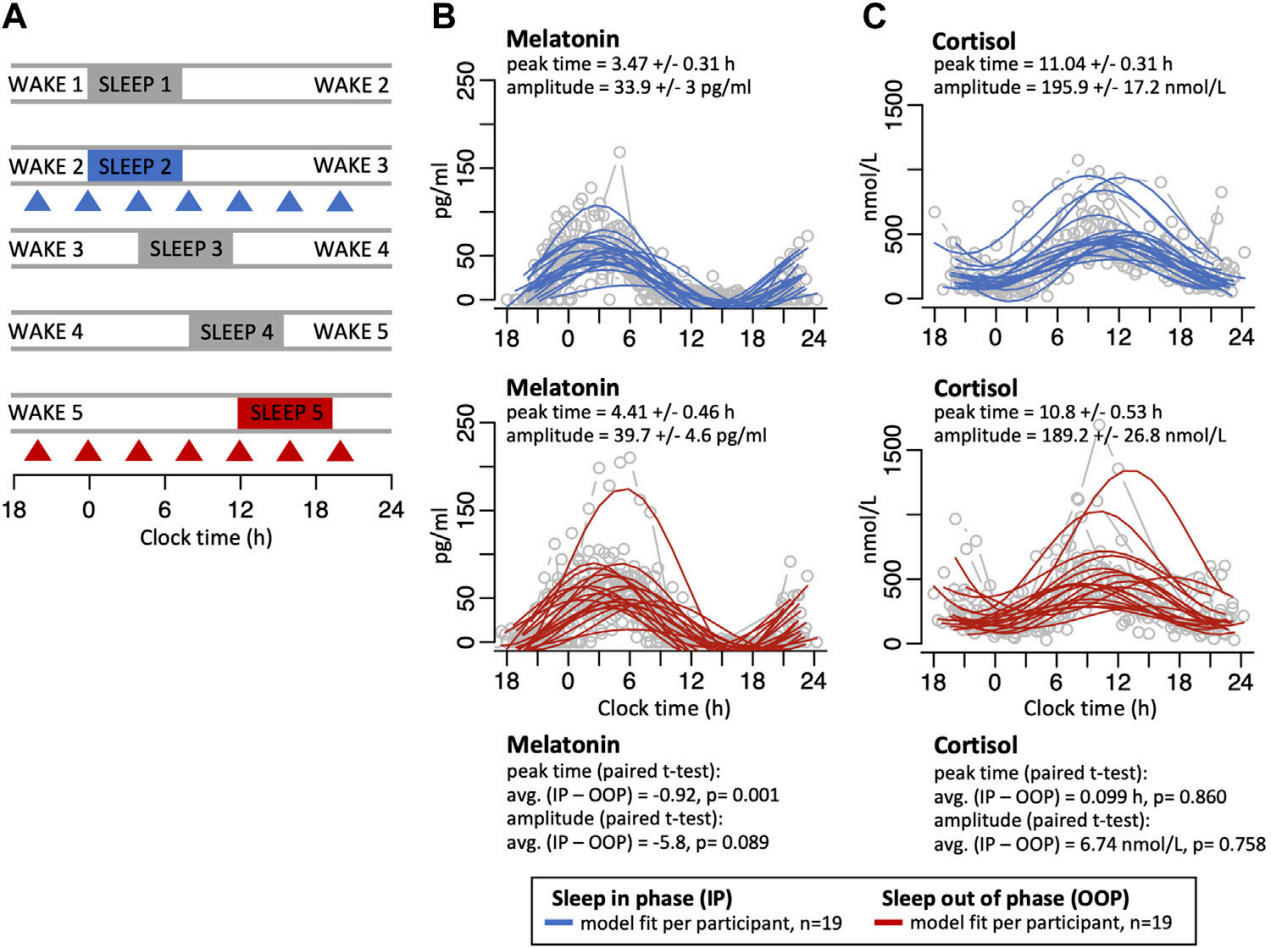
**(A)** After a normal 24-h sleep/wake period (wake1/sleep1), participants were scheduled to a proportionate 28-h day such that sleep occurred both in-phase with melatonin (wake2/sleep2, blue), and then 12-h out-of-phase with melatonin 3 days later (wake5/sleep5, red). During both conditions, 4-hourly whole-blood samples were collected to extract RNA for microarray-assessed genome-wide gene expression (triangles). Panels on the right show individual melatonin **(B)** and cortisol **(C)** profiles (grey lines/circles) with sine wave model best-fit curves for sleeping in-phase (solid blue lines) and sleeping out-of-phase (solid red lines). Peak times and amplitudes values for the best fit sine wave for each are shown (mean +/- SE), together with the average peak time difference and amplitude difference between the sleep in-phase (IP) and sleep out-of-phase (OOP) conditions with paired *t*-test *p* values.

In a previously reported study (Archer et al., 2014), 24 participants (mean ± SD of age, 26.3 ± 3.4 years; 11 males) underwent a forced desynchrony protocol which induces phase misalignment between rhythms driven by the SCN and the sleep–wake cycle. In this protocol the sleep/wake cycle, the dark/dim light cycle, and meals were scheduled for a 28-h day (Figure 1A) (see Hasan et al., 2012 for details) (Archer et al., 2014). Initially, i.e., during the second sleep/wake period of the protocol, sleep occurred in-phase with the central circadian clock in the SCN, as indexed by melatonin (measured from 2-hourly plasma samples, as described previously (Hasan et al., 2012)), whereas after 4 days, sleep and waking activities (i.e., light exposure, feeding) occurred 12 h out-of-phase with the melatonin rhythm, whose phase remained approximately the same in this dark/dim (<5 lux) light cycle. Throughout the protocol, sleep was assessed with polysomnography and total sleep time was 450.6 ± 7.6 min (mean ± SEM) when sleep occurred in phase, and then 446.5 ± 11.4, 401.7 ± 78.6, and 388 ± 17.6 min in the subsequent three delayed sleep episodes, respectively, demonstrating the small sleep loss that is known to occur in forced desynchrony protocols (Dijk and Czeisler, 1995).

During the sleeping in- and out-of-phase periods, 2-hourly plasma samples were collected from each participant to measure cortisol. Total cortisol was measured by radioimmunoassay in duplicate with an assay detection limit of 1.5 ± 0.5 nmol/L (mean ± SD), and intraassay coefficients of variation of 8.4%, 9.0%, and 7.5% for low (100.7 ± 8.5 nmol/L, mean ± SD), medium (656.4 ± 59.1 nmol/L), and high (1091.3 ± 82.0 nmol/L) quality control pools, respectively (Stockgrand Ltd, United Kingdom).

To measure melatonin and cortisol amplitude and phase, a curve of the form X = a+c sin(t+b) was fitted to each participant’s melatonin and cortisol time series using R’s lm ‘linear model’ method (Weisberg, 2011) on a linearised form of the equation [X = a+c sin(b) cos(t) + c cos(b) sin(t)] and a fixed period of 24 h. The peak times and amplitudes estimated from the sinewave model of the sleeping out-of-phase profiles were compared to the matched estimates from the sleeping in-phase profiles using the paired *t*-test.

During the sleeping in-phase and sleeping out-of-phase conditions, seven, 4-hourly blood samples were collected to measure the whole-blood transcriptome. Blood was collected into Paxgene Blood RNA tubes (BD Bioscience, New Jersey, United States), and mRNA was extracted and whole-genome transcriptome expression profiles were measured and analysed as previously described (Archer et al., 2014). In brief, total RNA was isolated using a PAXgene Blood RNA Kit and a QiaCube robot (Qiagen). Extracted RNA was quantified and the A260/280 nm and A260/230 nm ratios were determined using a NanoDrop ND1000 spectrophotometer. RNA quality was assessed using the Bioanalyzer 2100 (Agilent Technologies). Only RNA samples with an RNA Integrity Number (RIN) > 6 were subjected to microarray analysis; the majority of samples had a RIN score of >8. cRNA was synthesised and fluorescently labelled with Cy3-CTP from 100 ng of total RNA using the Low Input Quick Amp Labelling Kit (Agilent). Labelled cRNA (1.65 μg) was hybridised on a Whole Human Genome 4 × 44K v2 custom oligonucleotide microarray (G2514F, AMADID 026817; Agilent). Standard manufacturer’s instructions for one-colour gene-expression analysis were followed for labelling, hybridisation, and washing steps. Microarray data were submitted to the GEO archive (accession number GSE48113).

Effects of the protocol on the temporal transcriptome expression profiles were measured with linear mixed-model ANOVA (R, lmerTest, 3.1-3) (Kuznetsova AB and Christensen, 2017) with participant as a random effect and ‘sleep condition’ and ‘sample time’ as fixed effects, as described previously (Archer et al., 2014). ‘Sample time’ is the cortisol-phase-aligned sampling time sorted into eight equally sized bins to create categorical values. A significant ANOVA main effect identified an overall up- or down-regulation of expression between sleep conditions, whereas a significant interaction between ‘sleep condition’ and ‘sample time’ identified transcripts whose temporal expression profiles were affected by misaligned sleep. *p* values were corrected for multiple testing using the Benjamini Hochberg method (significant corrected *p* values <0.05) (Benjamini, 1995). For a full description of up- and down-regulated transcripts and rhythmic transcripts in each sleep condition and their associated biological processes and molecular functions, see Archer et al. (2014) for details.

For the analysis of the effect of misaligned sleep on the expression profiles of glucocorticoid signalling transcripts, we defined a set of 144 transcripts that code for 83 genes and was based upon the Qiagen glucocorticoid signalling pathway gene list (Qiagen, Hilden, Germany; Supplementary Table S1).

Gene ontology (GO) enrichment analysis for non-redundant biological processes and molecular functions was performed using WebGestalt 2019 (webgestalt.org; Liao et al., 2019) accessed in July 2021. The reference dataset used was the human Agilent 4x44k v2.

Enrichment of SP1 targets (as defined at https://maayanlab.cloud/Harmonizome/dataset/ENCODE+Transcription+Factor+Targets (12/9/2021)) was assessed using a hypergeometric test (phyper function). Each list of targets was refined to include only genes represented on the microarray platform described in Archer et al. (2014). The total set of genes represented on the microarray was set as the background.

Temporal coherence analyses. Glucocorticoid genes affected by sleeping out-of-phase (n = 44 genes/69 probes) were defined as those genes with a significant (BH *p* < 0.05) main effect of sleep condition (n = 17 genes/24 probes) or interaction of sleep condition and cortisol phase (*n* = 38 genes/57 probes); not-affected genes were those not significant in either main effect or interaction (*n* = 52 genes/75 probes). For each probe, the average expression across participants was calculated for each time point, generating a mean expression profile for sleeping in-phase and for sleeping out-of-phase. The R function cor.test was used to calculate the Pearson correlation coefficient and its statistical significance between the mean expression profile of *SP1* and each of the genes’ mean expression profile in the affected or not-affected group. We also performed circular cross-correlation analysis of each of the genes with *SP1* to investigate their temporal relations and how they may have changed during mistimed sleep. For each gene, the expression values at sampling time points 1 to 6 were averaged across participants (n = 19). The resulting mean expression temporal profile was z-scored. The circular cross-correlation was used to identify the time lag in hours at which the maximum correlation occurs between z-scored mean expression temporal profiles (Moller-Levet et al., 2022). Sampling time point 7 was not included in the calculations because it occurs at the same time of the first time point only 24 h apart and would not allow the time shifting to create a lag.

Please note that none of these analyses of the effects of the manipulations of sleep–wake on the transcriptome make any assumption about whether the expression profiles are rhythmic or not. The methods only assess whether there is a change in the temporal profile or the average expression level.

## 3 Results

### 3.1 Melatonin and cortisol rhythms remain largely unchanged when sleeping out-of-phase

In this study protocol, which simulates shift work, participants initially slept normally in phase with the central circadian clock, as indexed by melatonin rhythms, after which sleep was delayed by 4 h each day such that after 3 days sleep was mistimed by 12 h with respect to the central circadian clock (Figure 1A). Under this forced desynchrony protocol, the SCN-driven melatonin rhythm oscillates with an intrinsic period of ∼24.2 h and there was no significant change in the fitted mean amplitude values of the melatonin rhythms when sleep occurred in-phase and 12 h out-of-phase but the mean fitted peak time shifted by 0.92 h (*p* = 0.001) when sleep was mistimed (mean +/− SE fitted amplitude in-phase 33.9 +/− 3 pg/ml; mean fitted amplitude out-of-phase 39.7 +/− 4.6 pg/ml; mean +/- SE fitted peak time in-phase 3.47 +/− 0.31 h, mean fitted peak time out-of-phase 4.41 +/− 0.46 h; Figure 1B, individual melatonin profiles are plotted in Supplementary Figure S1). When plasma cortisol levels were measured, there were no major changes in the profile or amplitude values of the rhythms between the in-phase and out-of-phase conditions (mean +/- SE fitted amplitude in-phase 195.9 +/− 17.2 nmol/L; mean fitted amplitude out-of-phase 189.2 +/- 26.8 nmol/L; mean fitted peak time in-phase 11.04 +/- 0.31 h, fitted peak time out-of-phase 10.8 +/- 0.53 h; Figure 1C; individual cortisol profiles are plotted in Supplementary Figure S2).

### 3.2 Effect of sleeping out-of-phase on transcripts in glucocorticoid signalling pathways

**FIGURE 2:**
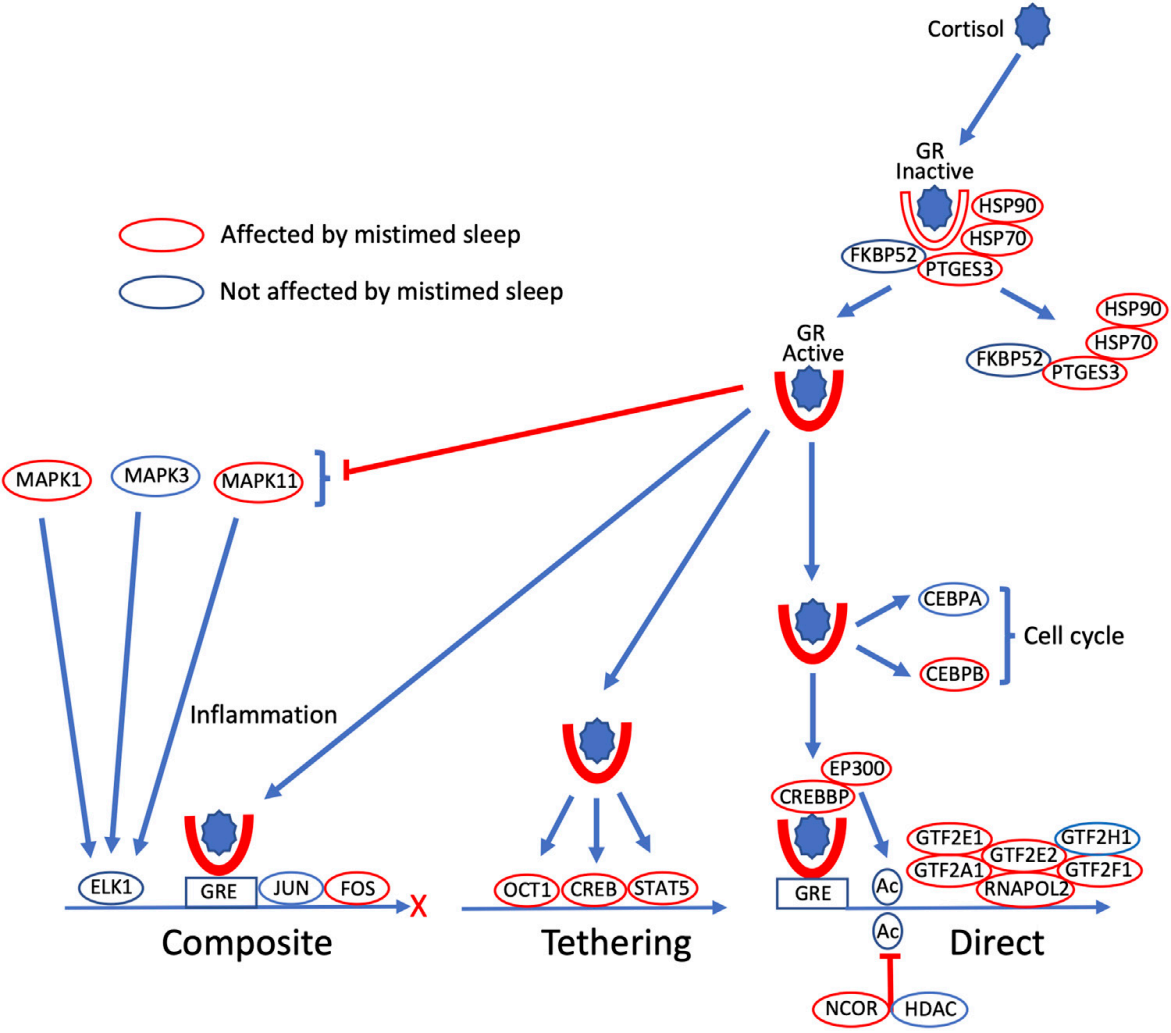
Glucocorticoid gene expression regulatory signalling pathways are disrupted by sleeping out-of-phase. Cortisol binds to cytoplasmic glucocorticoid receptors (GR), which become active when a set of bound chaperone proteins (FXBPS2, PTGES3, HSP70, HSP90) dissociate from GR. Active GR regulates gene expression in three major ways: GR binds to glucocorticoid response elements (GRE) in conjunction with other transcription factors (TFs; JUN, FOS) in composite regulation; GR promotes gene expression by positive interaction with other TFs (OCT1, CREB, STAT5) in regulation by tethering; in direct regulation, GR binding to GRE activates RNA polymerase two complexes (RNAPOL2, GTF2A1, GTF2E2, GTF2H1), which is also regulated by acetylation status (CREBBP, EP300 acetylation; NCOR, HDAC deacetylation). GR also has non-genomic signalling to regulate cell cycle components (CEBPA, CEBPB), and inhibits the production of inflammatory cytokines via MAP kinases (MAPK1, MAPK3, MAPK11). Within these signalling pathways, all encoding transcripts whose expression profile is disrupted by sleeping out-of-phase are highlighted by red ovals (unaffected encoding transcripts indicated by a blue oval).

**FIGURE 3:**
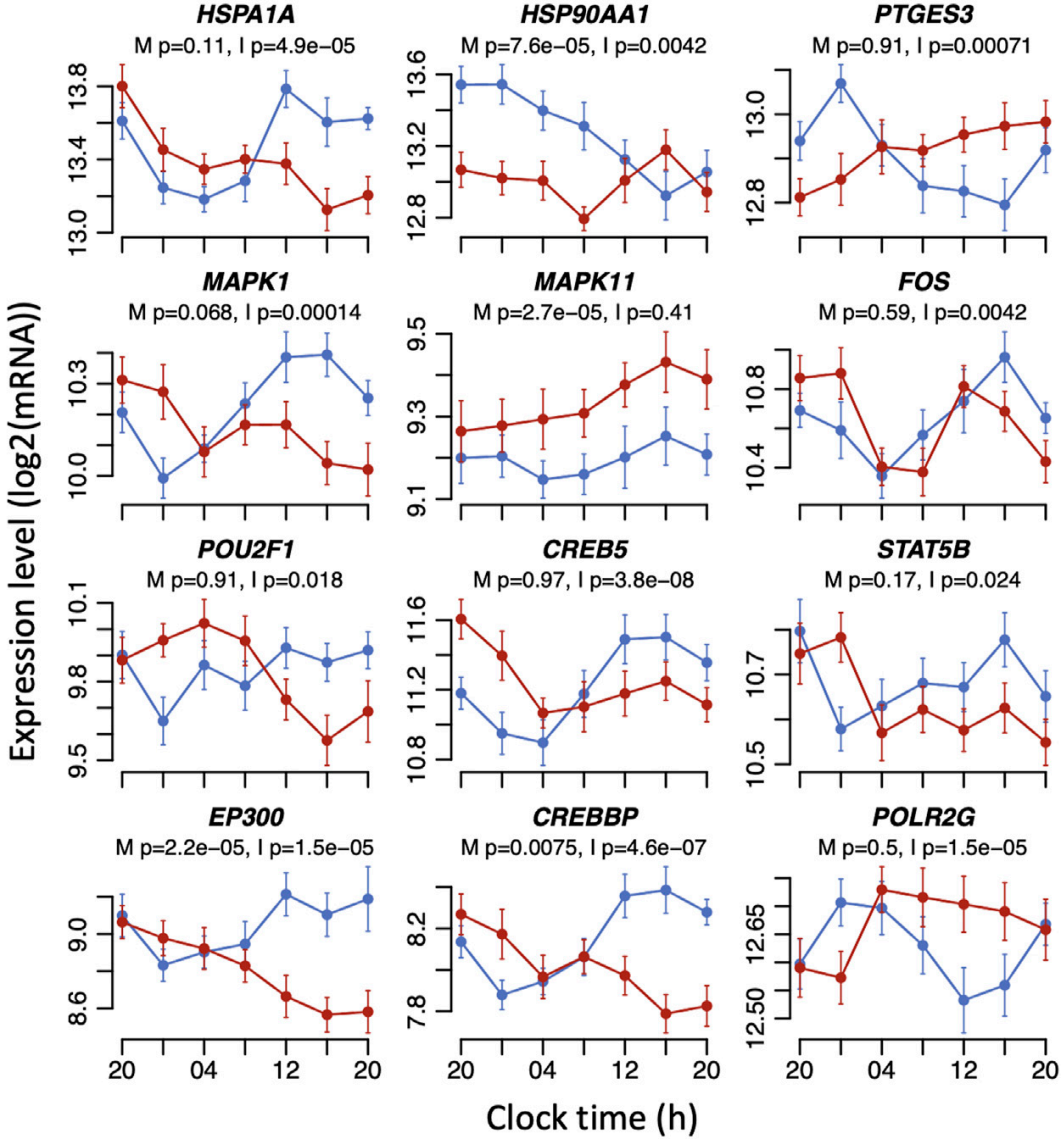
Example plots of disruption caused by sleeping out-of-phase to the expression profiles of glucocorticoid signalling transcripts. Mean RNA expression (log_2_ ± SE) profiles are plotted for sleeping in-phase (blue) and sleeping out-of-phase (red). Mixed model ANOVA BH-corrected *p* values are indicated for a main effect of sleep condition (M) or the interaction sleep condition x sample time (I). Please note that the main effects of sleep conditions or the interaction only imply that the mean expression levels and/or temporal profiles of transcripts have changed. The mixed model ANOVA does not rely on the assessment of the significance of circadian rhythmicity or rhythm parameters such as amplitude or phase.

The expression profiles of most of the transcripts that code for proteins in the glucocorticoid gene expression signalling pathways, including GR (*NR3C1*), are disrupted by sleeping out-of-phase (Figure 2; Supplementary Table S1). Twenty of these disrupted transcripts showed a significant interaction for sleep condition and sample time, indicating that the temporal organisation of their expression profiles had been affected by sleep out-of-phase (for example plots, see Figure 3). Some transcripts also showed a main effect of sleep condition, e.g., when sleeping out-of-phase *HSP90AA1* and *EP300* had reduced expression levels, while *MAPK11* had increased expression (Figure 3).

Glucocorticoid signalling pathways contain many more elements than those depicted in Figure 2. We defined a set of 144 transcripts (associated with 83 genes) that are involved in glucocorticoid signalling pathways (Supplementary Table S1). Of these, 12 transcripts showed just a main effect of sleep condition, 46 transcripts showed just an interaction between sleep condition and sample time, and 11 transcripts had both a main effect and interaction (see Supplementary Table S1). Thus, 48% of the signalling transcripts were affected by mistimed sleep. Some of the most significantly affected transcripts included the transcriptional repressor *NCOR1* (main effect *p* = 1.87e-05, interaction *p* = 0.0071), protein kinase *MAPK11* (main effect *p* = 2.72e-05), the heat shock glucocorticoid chaperone proteins *HSP90AA1* (main effect *p* = 7.63e-05, interaction *p* = 0.0042) and *HSPA1A* (interaction *p* = 4.87e-05), cAMP response element-binding protein transcription factor *CREB5* (interaction *p* = 3.78e-08), and *CREBBP* (main effect *p* = 0.0074, interaction *p* = 4.61e-07) and *EP300* (main effect *p* = 2.18e-05, interaction *p* = 1.48e-05), which function together as histone acetyltransferase transcriptional regulators and also bind CREB.

### 3.3 Effect of sleeping out-of-phase on transcripts induced by dexamethasone in mouse adipocytes and human blood

**FIGURE 4:**
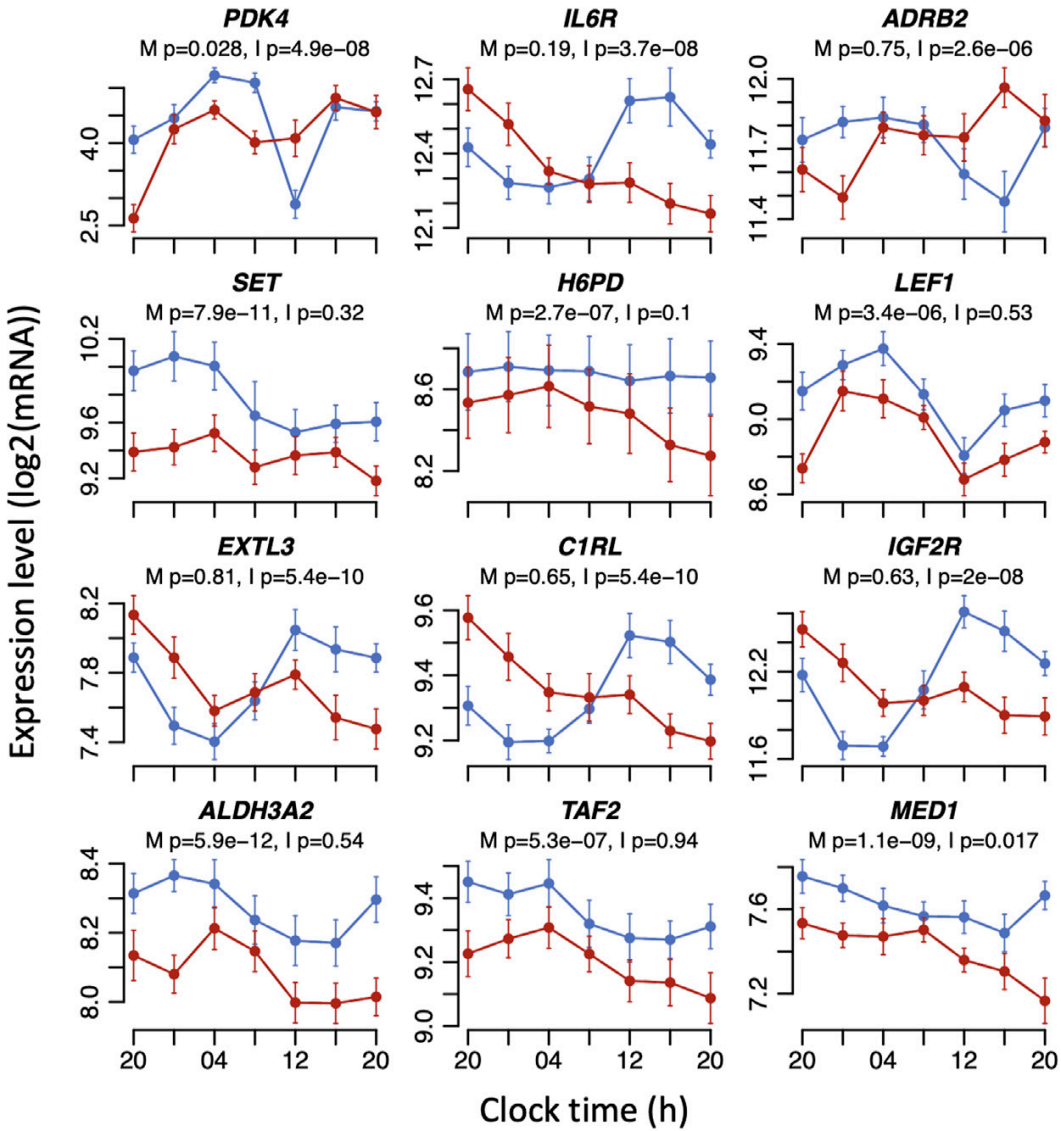
Example expression plots for mouse adipocyte (top six) and human whole blood (bottom six) dexamethasone-induced transcripts that are significantly affected by sleeping out-of-phase in the mistimed sleep dataset. Mean RNA expression (log_2_ ± SE) profiles are plotted for sleeping in-phase (blue) and sleeping out-of-phase (red). Mixed model ANOVA BH-corrected *p* values are indicated for a main effect of sleep condition (M) or the interaction sleep condition x sample time (I). Please note that the main effects of sleep conditions or the interaction only imply that the mean expression levels and/or temporal profiles of transcripts have changed. The mixed model ANOVA does not rely on the assessment of the significance of circadian rhythmicity or rhythm parameters such as amplitude or phase.

Previous studies have identified sets of genes whose expression is regulated by glucocorticoid. For example, Yu et al. (2010) treated mouse adipocyte cells in culture with dexamethasone to define a set of 619 glucocorticoid-induced genes. That set maps to 868 orthologous transcripts within our gene expression matrix for this study, of which 254 transcripts (29%) showed either a main effect of sleep condition or an interaction between sleep condition and sample time (Supplementary Table S2; Figure 4). Thus, around one-third of glucocorticoid-regulated genes expressed in both adipocytes and blood are also affected by sleeping out-of-phase with the central SCN clock. Enrichment analysis of gene ontology (GO) terms in the affected 254 transcripts identified biological processes and molecular functions that included ‘fat cell differentiation’ (*p* = 2.349e-8, FDR = 0.00002), ‘regulation of lipid metabolic process’ (*p* = 1.800e-6, FDR = 0.00101), ‘steroid metabolic process’ (*p* = 8.844e-06, FDR = 0.002651), ‘response to nutrient levels’ (*p* = 0.00038, FDR = 0.02807), and ‘response to oxidative stress’ (*p* = 0.00042, FDR = 0.02807).

A study by Menke et al. (2014) investigated dexamethasone-induced gene expression in human whole blood in relation to job-related exhaustion. In the control group for that study, dexamethasone induced the expression of 6,219 transcripts, which mapped to 6,193 transcripts in our dataset. Of those, 3,072 transcripts showed either a main effect of sleep condition or an interaction between sleep condition and sample time in response to mistimed sleep, representing 50% of transcripts (Supplementary Table S3; Figure 4). This larger overlap compared to the mouse dexamethasone-induced transcripts presumably reflects the direct comparison between two human whole blood transcriptomes. Enrichment analysis of GO terms in the affected human transcripts revealed top ten ontology terms that included ribonucleotide complex biogenesis (*p* = 0, FDR = 0), translation initiation (*p* = 0, FDR = 0), protein localisation to endoplasmic reticulum (*p* = 1.1102e-16, FDR = 2.5135e-14), and neutrophil-mediated immunity (*p* = 3.9684e-11, FDR = 4.4922e-9). Thirty-three percent of the significantly affected mouse dexamethasone-induced transcripts overlapped with significantly affected dexamethasone-induced human transcripts.

### 3.4 Correlation between expression profiles of glucocorticoid signalling transcripts and the transcription factor SP1

**FIGURE 5:**
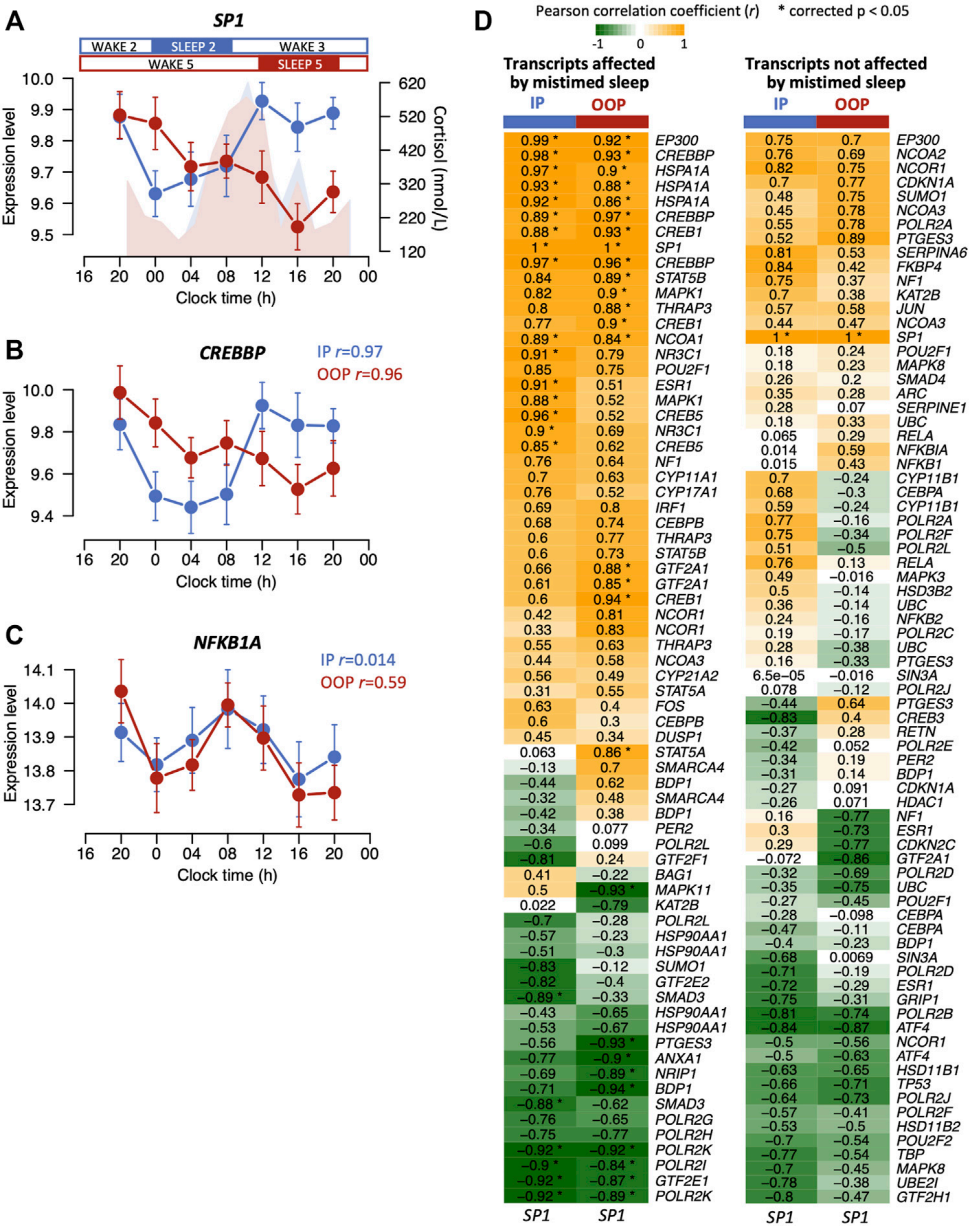
**(A)** Mean expression levels (log_2_ ± SE) for *SP1* across 24 h when sleeping in-phase (blue) and when sleep was 12 h out-of-phase (red). Plasma cortisol profiles (nmol/L) are shown for in-phase (blue) and out-of-phase sleep (red). The mean expression profiles of some glucocorticoid signalling transcripts such as *CREBBP* **(B)** correlate with *SP1* when sleep occurred in-phase (blue) but also when sleep occurred out-of-phase (red), whereas no signalling transcripts unaffected by mistimed sleep remained correlated with *SP1* when sleep occurred out-of-phase (e.g., *NFKB1A*) **(C)**. For both **(B,C)**, correlation r values are indicated for in-phase (IP) and out-of-phase (OOP) sleep. **(D)** Heatmaps for correlation r values between the mean expression profiles of glucocorticoid signalling transcripts and the *SP1* mean expression profile when sleep occurred in-phase (IP) and out-of-phase (OOP). Correlation heatmaps are shown for transcripts affected (left panel) and not affected (right panel) by mistimed sleep. Asterisks in the heatmap cells indicate significant correlations. Note: there may be multiple transcripts (probes) present for the same gene.

In our previous analysis of this dataset, we found that a transcription factor *SP1* was significantly affected by mistimed sleep (Archer et al., 2014) (Figure 5A). SP1 is a transcription factor that binds to the promoters of many genes regulating many cellular processes and we previously showed in an interaction network analyses that SP1 interacted with the largest number of other transcripts affected by mistimed sleep (Archer et al., 2014). Analysis of the glucocorticoid signalling transcripts (GSTs) and the mouse and human dexamethasone-induced transcripts whose expression profiles were affected by mistimed sleep showed a significant enrichment for SP1 transcription factor binding sites (*p* = 2.51E-12, *p* = 6.45E-40, and *p* < 2.23E-308, respectively, presence or absence of SP1 site indicated 1 or 0, respectively, in Supplementary Tables S1-S3). The disruption to the *SP1* expression profile due to mistimed sleep resembles the disruption observed in many of the GSTs and dexamethasone-induced transcripts affected by mistimed sleep. To investigate this relationship further, we proceeded in two ways. We first calculated the correlations between the mean expression profile of *SP1* and the mean expression profiles of GSTs affected and not affected by mistimed sleep for both sleeping in- and out-of-phase (Figure 5). Of the 69 transcripts affected by mistimed sleep, the expression profiles of 21 were significantly correlated with expression profiles of *SP1* when sleeping in-phase, and 26 were correlated when sleeping out-of-phase. By contrast, of the 75 transcripts that were not affected by mistimed sleep, none were correlated with *SP1* when sleeping in-phase and out-of-phase. Thus, expression profile of many of the GSTs that are affected by mistimed sleep are associated with expression levels of *SP1*.

**FIGURE 6:**
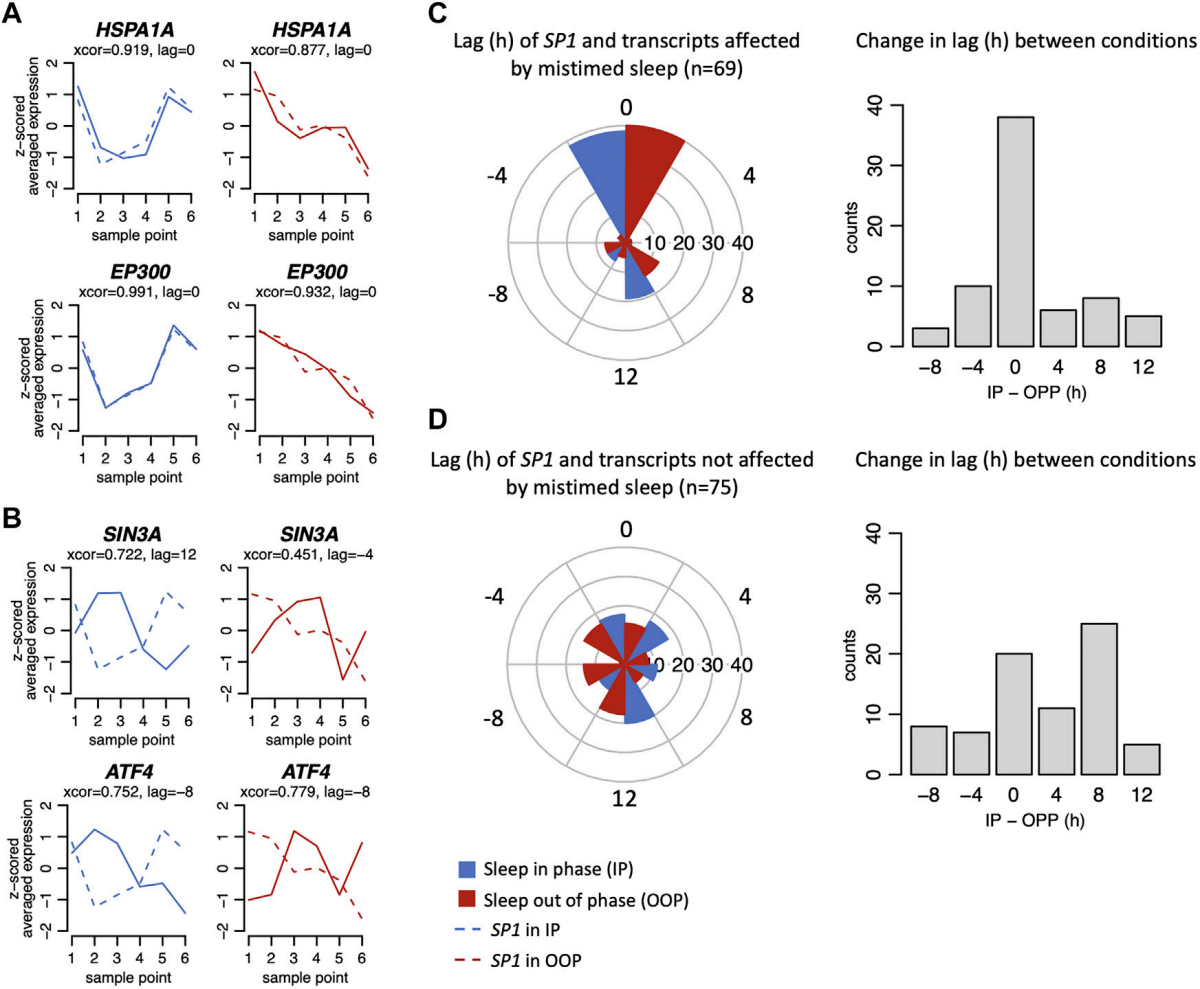
Example plots of expression profiles (z-scored) when in-phase (blue) and out-of-phase (red) for glucocorticoid signalling transcripts affected **(A)** and not affected **(B)** by mistimed sleep. Example plots (solid lines) are shown overlying plots for *SP1* (dashed lines). For each plot, the results from the circular cross-correlation analysis are provided with the lag value indicating the timing difference between the example transcript and *SP1*. **(C)** Left. Polar plots show the lag in hours *SP1* and transcripts affected by mistimed sleep when sleep occurred in-phase (IP) and out-of-phase (OOP), and on the right, the difference of those lag values between IP and OOP sleep conditions. **(D)** As for **(C)**, but for signalling transcripts not affected by mistimed sleep.

We next investigated the temporal relationships between the 24 h expression profiles of GSTs and *SP1*. Circular cross-correlation analysis showed that the temporal expression profiles of the majority of GSTs that were affected by mistimed sleep correlated with the expression profile of *SP1* both when sleep occurred in-phase and out-of-phase and that the lag did not change when sleeping out-of-phase (Figures 6A,C). However, this was not the case for the signalling transcripts that were not affected by mistimed sleep. For these transcripts, the range of temporal relationships was broad when sleeping in-phase (Figures 6B,D) and these temporal relationships showed a wide range of changes when comparing in-phase vs out-of-phase (Figures 6C,D). Thus, in terms of both mean expression profiles and expression timing, a larger number of GSTs that are affected by mistimed sleep are significantly correlated with the expression of *SP1*. To investigate further the regulation of GSTs by SP1, we constructed an interaction network using STRING (string-db.org, v11.5, high-confidence evidence setting; (Szklarczyk et al., 2021)) with the total list of 144 GSTs (Supplementary Table S1) together with SP1. The high-confidence edges (connections between interacting protein nodes) were then imported into Cytoscape (cytoscape.org, v3.8.2; Shannon et al., 2003) and arranged in two layers representing direct or indirect interaction with SP1 (Supplementary Figure S3). The resulting network clearly shows a high degree of both direct and indirect known interactions with SP1. In the figure, we also indicate which protein-encoding transcripts are known from ENCODE to contain promoter SP1 binding sites.

## 4 Discussion

In a forced desynchrony protocol in dim light, where after three 28-h days the sleep/wake cycle was 12 h out-of-phase with the central SCN clock, we showed that, like the melatonin rhythm, the amplitude and timing of the plasma cortisol rhythm remained largely unchanged. This is entirely consistent with the cortisol rhythm being driven by the central circadian clock and is also consistent with previous forced desynchrony data (Scheer et al., 2009; Dijk et al., 2012; McHill et al., 2018). It should be noted that in this protocol the rest/activity cycle is desynchronised so that in addition to sleep, other cycles such as feeding/fasting and light/dark are also mistimed. Previously, we showed that the same forced desynchrony protocol resulted in a reduction of rhythmic whole-blood transcripts from 6.4% when sleeping in-phase to 1.0% when sleeping out-of-phase (Archer et al., 2014). We also showed that more than one-third of all transcripts showed a significant interaction between sleep condition and sample time, indicating that their temporal expression profiles were affected by sleeping out-of-phase. Here, we focussed on glucocorticoid signalling transcripts that were included in the previous dataset and asked the question whether the previously observed disruption to expression profiles caused by forced desynchrony was also seen in transcripts in glucocorticoid signalling pathways in blood, even though plasma cortisol rhythms remained unchanged. We decided not to apply methods to detect 24 h rhythmicity, because even though many of the profiles appear rhythmic, whether they are rhythmic or not is irrelevant to the question we address here, which was what are the effects of mistimed sleep on cortisol and how does this compare to effects of mistimed sleep on glucocorticoid signalling transcripts. We found that the temporal expression profiles of almost half of all GSTs in blood were affected by sleeping out-of-phase with the central clock. Furthermore, in datasets of adipocyte mouse transcripts and human whole blood transcripts shown to be induced by dexamethasone, 29% and 50% of transcripts, respectively, were affected by mistimed sleep in the current dataset. By combining bioinformatics and temporal coherence analyses, we also showed that a significant number of affected transcripts contain binding motifs for the transcription factor SP1 and that the amplitude and temporal expression profiles of many GSTs correlate with *SP1* when sleep is both in-phase and mistimed.

The glucocorticoid signalling pathways that we have focussed on in this study all initiate with the binding of cortisol to glucocorticoid receptors. Therefore, an intriguing question is why are many of the transcripts coding for the downstream glucocorticoid signalling molecules disrupted by mistimed sleep when the cortisol rhythm is not; what are the underlying mechanisms? If the glucocorticoid signalling transcripts were regulated solely by cortisol, then we would expect their expression profiles to remain locked to the cortisol rhythm and be unchanged with mistimed sleep. The fact that they were disrupted must indicate that their expression is regulated by other factors, either independent or in addition to cortisol. Likewise, we would expect the transcripts for genes whose expression was induced by dexamethasone in mouse adipocytes and human blood to be regulated by cortisol *in vivo*. If those transcripts were solely regulated by cortisol, then we would not necessarily expect them to be disrupted by mistimed sleep. Together, these findings must mean that in addition to being regulated by cortisol, the rhythmic expression of those transcripts is also co-regulated by other transcription factor elements, or RNA modifying elements, that are disrupted by mistimed sleep.

These observations imply that there are sets of transcripts whose expression is driven by circadian factors (e.g., cortisol), sleep–wake-dependent factors (as yet undetermined), or a combination of both. Indeed, a previous study in mice has shown that brain transcripts affected by sleep deprivation can be subdivided into those that are only affected in the presence of corticosterone and a smaller subset that remain affected by sleep deprivation when corticosterone is removed by adrenalectomy (Mongrain et al., 2010). Maret et al. also showed that a majority of diurnally rhythmic brain transcripts become arrhythmic when sleep deprivation occurred around the clock implying that most of the rhythmic transcripts were in fact sleep–wake driven (Maret et al., 2007).

What could be the sleep–wake-regulated mechanism underlying the observed transcriptome disruption? In our dataset, *SP1* was significantly disrupted by mistimed sleep (Figure 5A) and we have shown here that SP1 sites are enriched in the promoters of many glucocorticoid signalling transcripts and mouse and human transcripts induced by dexamethasone. SP1 is also known to interact directly or indirectly with 82 of the 83 glucocorticoid signalling genes from Supplementary Table S1 (Supplementary Figure S3). SP1 is also known to interact with some of the most significantly disrupted dexamethasone-induced transcripts analysed here; a subunit of the transcription regulator mediator complex MED1 (Figure 4) is required for SP1 activation (Taatjes et al., 2002), and TAF2 (Figure 4) is a component of the transcription factor TFIID required for transcription complex assembly and activation of SP1 (Ryu et al., 1999).

*SP1* normally has a day peak that is suppressed when sleep is mistimed (Figure 5A) and has expression profiles that are very similar to the example plots in Figure 3 (see also Figure 5B and Figure 6A), e.g., CREBBP and EP300, with which SP1 interacts in the rhythmic regulation of gene expression (Koike et al., 2012). Indeed, we have shown that the majority of the glucocorticoid signalling transcripts that were affected by mistimed sleep have temporal expression profiles whose mean levels and timing correlate with those of SP1 in both in-phase and out-of-phase sleep conditions. Thus, suppression of biological day-peaking SP1 when sleep is mistimed could represent the sleep–wake drive that becomes in conflict with the day-peaking circadian drive from cortisol and influences the expression of other transcripts driven by SP1.

Evidence clearly suggests that SP1 could contribute to the regulation of expression of glucocorticoid-driven transcripts and their disruption during mistimed sleep, but what regulates the expression and action of SP1? The expression of *SP1* is regulated by several transcription factors including STAT3 and RAD51, which are both affected by mistimed sleep here, and also by SP1 itself (Tapias et al., 2008). Another glucocorticoid signalling component the heat shock protein HSP90AA1 is highly affected by mistimed sleep (Figure 3) and also regulates SP1 activity such that inhibition of HSP90AA1 reduces SP1-mediated gene expression (Liu et al., 2021). Thus, these mechanisms taken together may provide a framework by which mistimed sleep affects the expression of *SP1*, the activity of SP1 protein as a transcription factor, and subsequent feedback to regulate its own expression.

The transcriptional activity of SP1 is influenced by phosphorylation, e.g., phosphorylation of SP1 regulates EP300 activity (Swift et al., 2021). SP1 activity is increased by phosphorylation by MAPK1 (Hu et al., 2017), whose expression levels were significantly reduced during mistimed sleep (Figure 3). There is growing evidence that accumulating levels of protein phosphorylation during wake promotes sleep and MAP kinases are involved in this process (Ode and Ueda, 2020). The transcription factor MEF2C regulates synaptic function and plasticity in response to sleep loss in a phosphorylation-dependent manner and is a key regulator of sleep function (Bjorness et al., 2020). Of interest, MEF2C and SP1 have been shown to cooperate in transcriptional regulation (Jiang et al., 2016), and the phosphorylation status of both is also regulated by the phosphatase calcineurin (Santini et al., 2001). A protein interaction network centred on MEF2C includes SP1, and also several MAP kinases, in addition to other proteins of note such as EP300, CREBBP, and CLOCK (Supplementary Figure S4). Therefore, we provide both experimental and theoretical evidence to support the role of SP1 in the sleep-driven regulation of glucocorticoid signalling.

It should be acknowledged that we report gene expression data from whole blood which includes RNA from all cell types and diurnal rhythms in the number of these cells have been described (Ackermann et al., 2012). Neutrophils are the most numerous cell type (∼60%) and are made during the day and cleared at night. Thus, much of the temporal organisation in gene expression that we observe may be largely driven by neutrophils and we do not know how this temporal organisation among different cell types becomes desynchronised with mistimed sleep. In addition, these data are restricted to the transcriptome and we cannot say what is happening at the level of the proteome. However, they nevertheless emphasise the potential for significant disruption to glucocorticoid signalling pathways and glucocorticoid-induced gene expression that could lead to subsequent impact on multiple physiological processes regulated by cortisol.

During shift work, cortisol rhythms remain largely unchanged and thus become misaligned with the rest–activity cycle. This circadian misalignment is associated with a wide range of negative health impacts. Cortisol regulates many physiological processes, such as immune function, glucose metabolism, insulin secretion, cardiovascular function, and circadian misalignment of the cortisol rhythm may underlie many of the adverse health effects linked with shift work. In addition to this circadian misalignment of cortisol, we have also presented novel findings of disruption by mistimed sleep to glucocorticoid signalling transcripts and transcripts whose expression is induced by dexamethasone. We also provide compelling evidence that much of the transcriptomic disruption caused by mistimed sleep is correlated with disruption to the expression of the transcription factor *SP1* and may be driven by its activity in a sleep-dependent manner.

## Data Availability

The data presented in the study are deposited in the GEO repository, accession number GSE48113.
