# Supplementary material for: Mistimed sleep and waking activity in humans disrupts glucocorticoid signalling transcripts and SP1, but not plasma cortisol rhythms
Source: Front Physiol. 2022 Aug 17;13:946444. doi: 10.3389/fphys.2022.946444 (PMC9428761; doi:10.3389/fphys.2022.946444)
Supplement: Supplementary file 3 [file DataSheet1.pdf]

Supplementary Material

1 Supplementary Figures

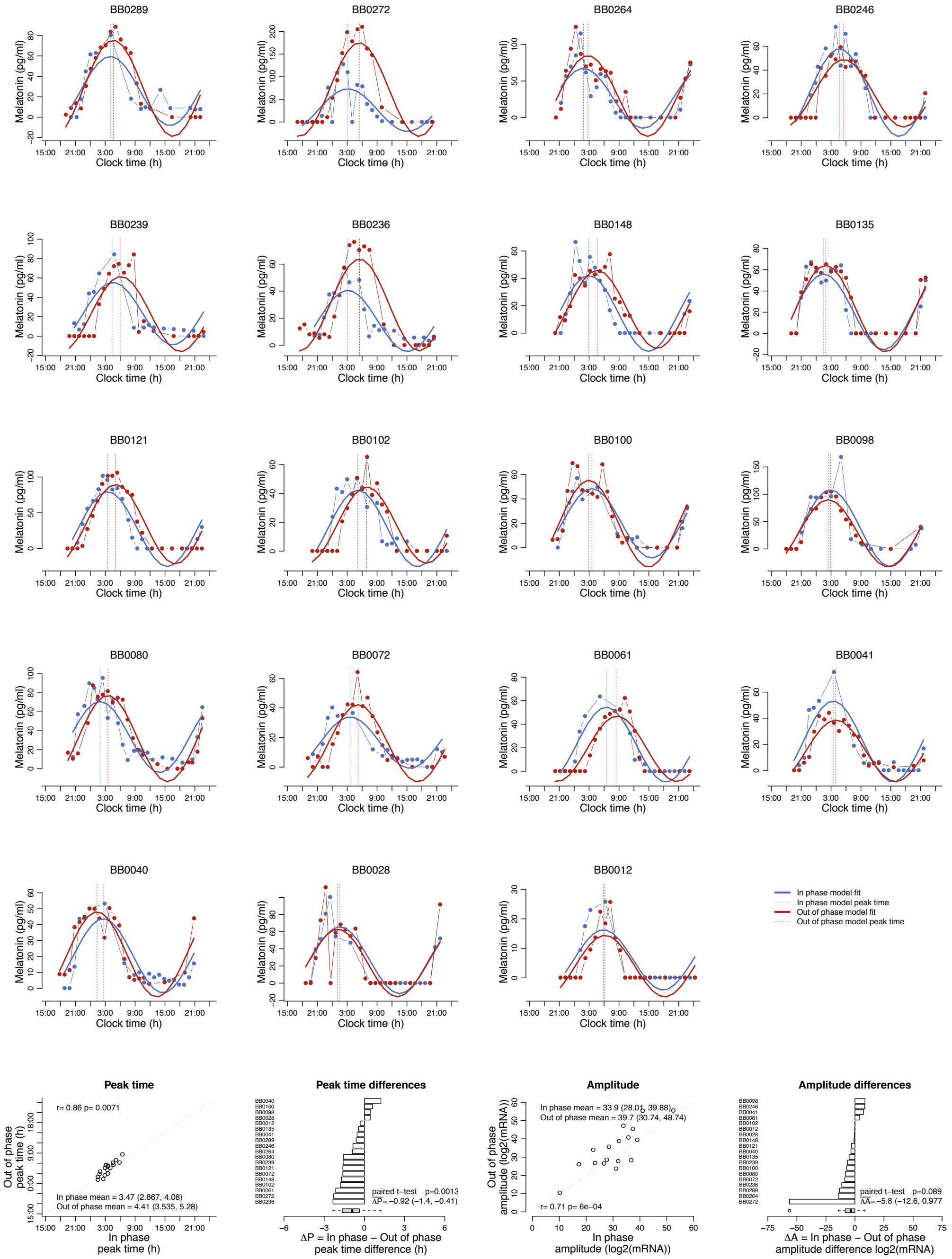

Supplementary Figure 1. Individual participant melatonin profiles plotted as pg/ml against relative clock time for in-phase (blue) and out-of-phase (red) conditions. Individual peak times and amplitudes are plotted for in-phase against out-of-phase, together with individual peak time and amplitude differences for in-phase minus out-of-phase.

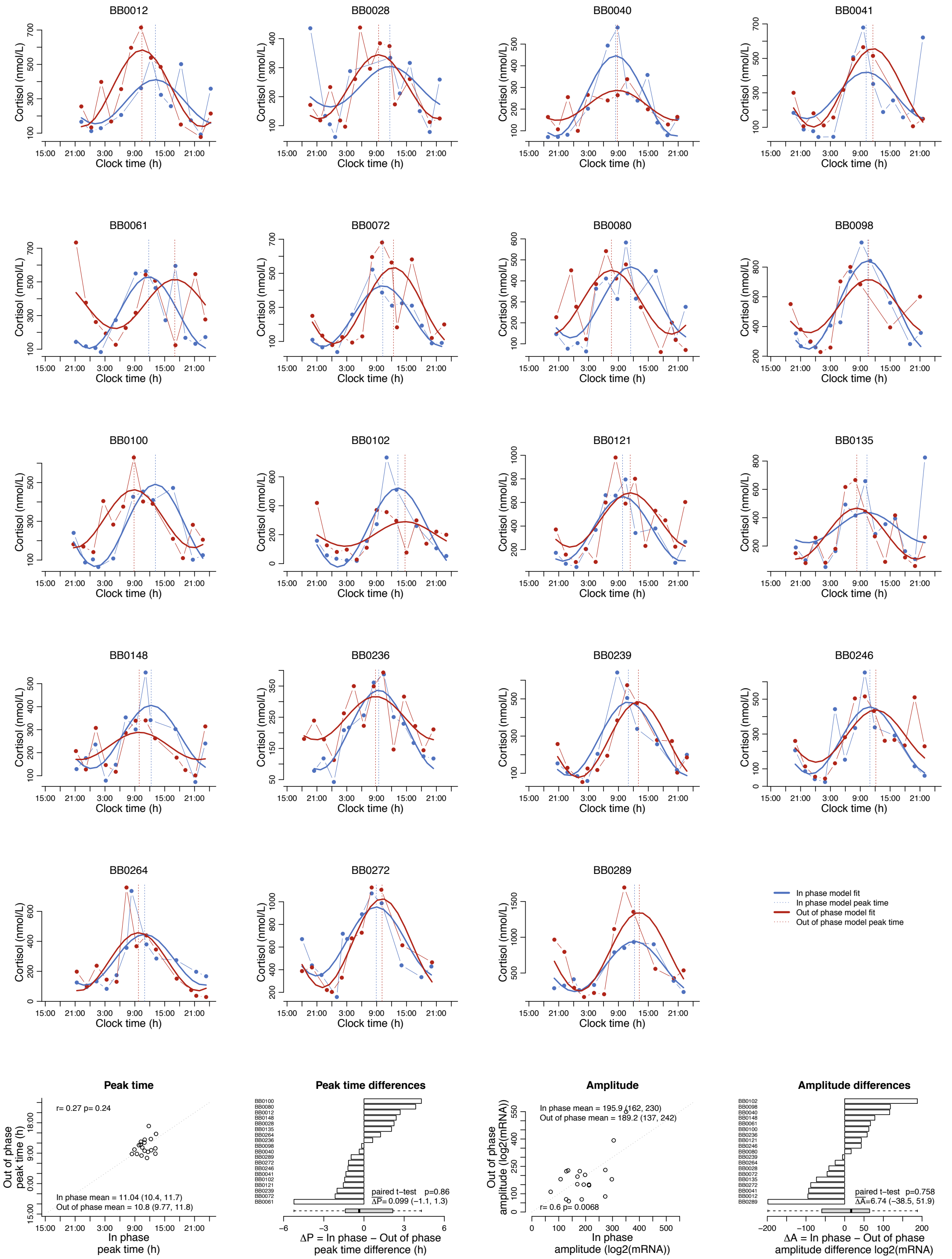

Supplementary Figure 2. Individual participant cortisol profiles plotted as nmol/L against relative clock time for in-phase (blue) and out-of-phase (red) conditions. Individual peak times and amplitudes are plotted for in-phase against out-of-phase, together with individual peak time and amplitude differences for in-phase minus out-of-phase.

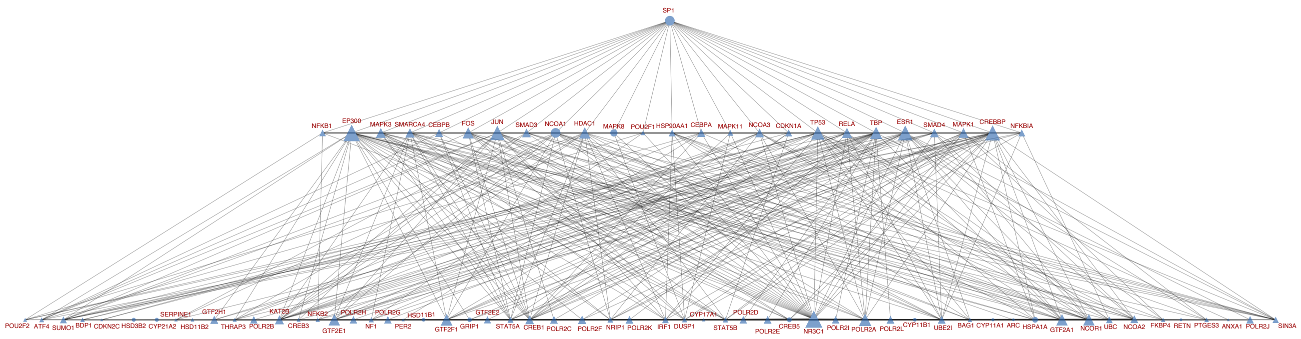

Supplementary Figure 3. Glucocorticoid signaling transcripts interaction network. Glucocorticoid signaling transcripts as reported in Supplemental Data File 1. Node shape: triangle indicates a gene that has an upstream SP1 binding site as identified in the ENCODE database, and an ellipse indicates a gene not known to have an SP1 binding site. Node size correlates with the number of edges (interactions) the node has within the presented network.

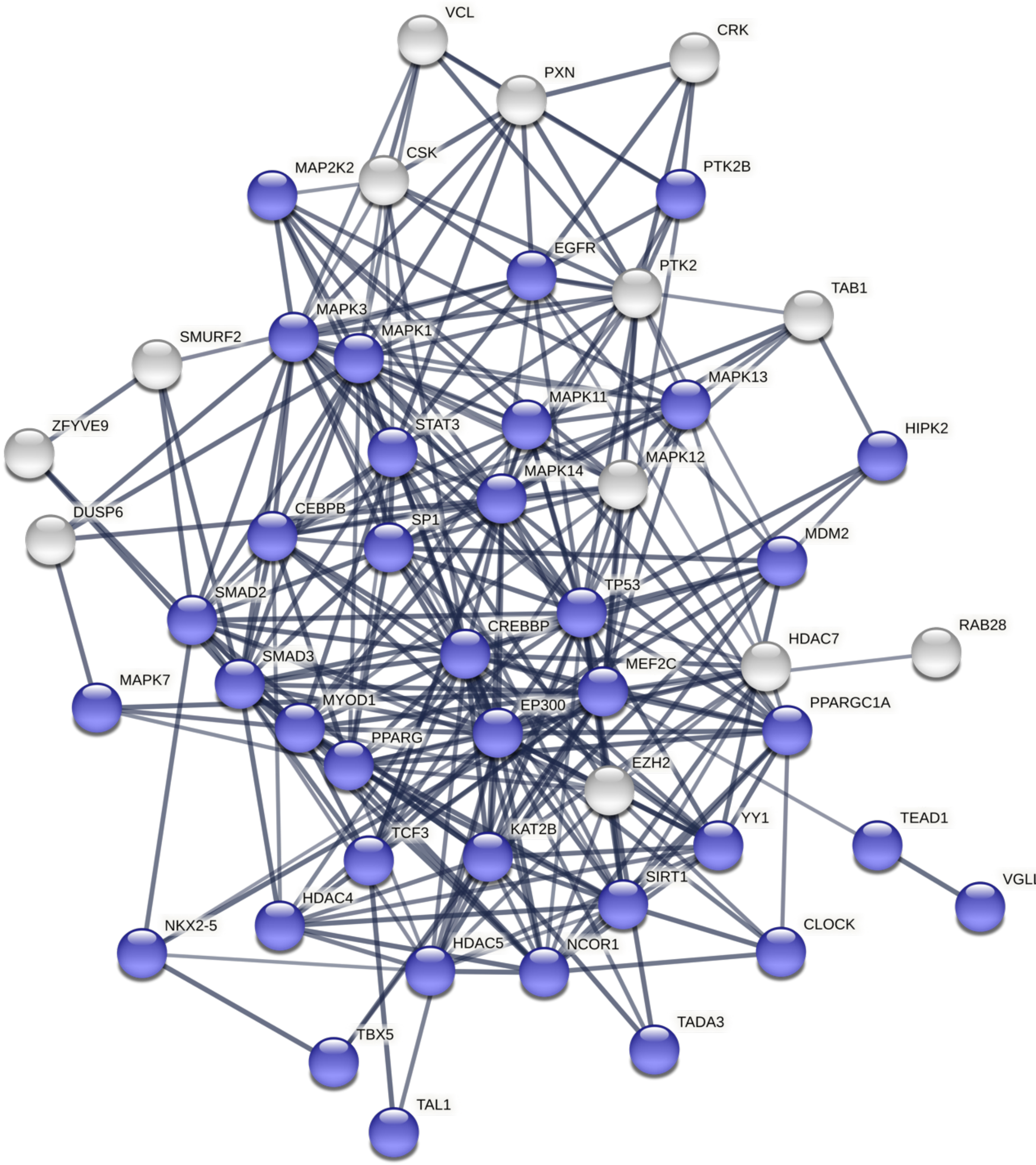

Supplementary Figure 4. Protein interaction network centered on MEF2C, created with STRING (v11.5, string-db.org) using high evidence confidence (minimum interaction score 0.70). Edge line thickness indicates strength of evidence. Blue-coloured nodes are enriched for the Gene Ontology (GO) term ‘Positive regulation of gene expression’ (GO: 0010628; FDR = 3.329e-20).
